# Boron neutron capture therapy plus bevacizumab versus bevacizumab alone in recurrent glioblastoma: A propensity score–matched analysis

**DOI:** 10.1093/noajnl/vdag013

**Published:** 2026-01-23

**Authors:** Shuo-Fu Chen, Yi-Yen Lee, Chun-Yu Liu, Chun-Fu Lin, Sanford P C Hsu, Feng-Chi Chang, Chih-Chun Wu, Shih-Chieh Lin, Ko-Han Lin, Jia-Cheng Lee, Jinn-Jer Peir, Fong-In Chou, Hiroki Tanaka, Yu-Ming Liu, Yi-Wei Chen

**Affiliations:** Department of Heavy Particles & Radiation Oncology, Taipei Veterans General Hospital, Taipei, Taiwan; Department of Neurosurgery, Taipei Veterans General Hospital, Taipei, Taiwan; School of Medicine, National Yang Ming Chiao Tung University, Taipei, Taiwan; School of Medicine, National Yang Ming Chiao Tung University, Taipei, Taiwan; Department of Oncology, Taipei Veterans General Hospital, Taipei, Taiwan; Division of Transfusion Medicine, Department of Medicine, Taipei Veterans General Hospital, Taipei, Taiwan; Department of Neurosurgery, Taipei Veterans General Hospital, Taipei, Taiwan; School of Medicine, National Yang Ming Chiao Tung University, Taipei, Taiwan; Department of Neurosurgery, Taipei Veterans General Hospital, Taipei, Taiwan; School of Medicine, National Yang Ming Chiao Tung University, Taipei, Taiwan; School of Medicine, National Yang Ming Chiao Tung University, Taipei, Taiwan; Department of Radiology, Taipei Veterans General Hospital, Taipei, Taiwan; School of Medicine, National Yang Ming Chiao Tung University, Taipei, Taiwan; Department of Radiology, Taipei Veterans General Hospital, Taipei, Taiwan; Department of Pathology and Laboratory Medicine, Taipei Veterans General Hospital, Taiwan; Department of Nuclear Medicine, Taipei Veterans General Hospital, Taipei, Taiwan; Department of Heavy Particles & Radiation Oncology, Taipei Veterans General Hospital, Taipei, Taiwan; Department of Medical Imaging and Radiological Technology, Yuanpei University of Medical Technology, Hsinchu City, Taiwan; Department of Nuclear Science & Technology Development, National Tsing-Hua University, Hsinchu City, Taiwan; Department of Nuclear Science & Technology Development, National Tsing-Hua University, Hsinchu City, Taiwan; Institute for Integrated Radiation and Nuclear Science, Kyoto University, Kumatori, Osaka, Japan; Department of Heavy Particles & Radiation Oncology, Taipei Veterans General Hospital, Taipei, Taiwan; School of Medicine, National Yang Ming Chiao Tung University, Taipei, Taiwan; Department of Heavy Particles & Radiation Oncology, Taipei Veterans General Hospital, Taipei, Taiwan; School of Medicine, National Yang Ming Chiao Tung University, Taipei, Taiwan; Department of Medical Imaging and Radiological Technology, Yuanpei University of Medical Technology, Hsinchu City, Taiwan

**Keywords:** bevacizumab, boron neutron capture therapy, glioblastoma, recurrent, re-irradiation

## Abstract

**Background:**

Boron neutron capture therapy (BNCT) presents a targeted re-irradiation strategy for recurrent glioblastoma. However, its survival benefit remains unclear, as few studies have compared the addition of BNCT to bevacizumab (BEV) with BEV alone or other salvage approaches.

**Methods:**

We retrospectively analyzed 171 adults with recurrent glioblastoma: 116 treated with BEV ± chemotherapy (control group) and 55 treated with BNCT plus BEV ± chemotherapy (BNCT group). To improve comparability, propensity score matching was performed using age, performance status, recurrent tumor volume, recurrence-to-salvage-treatment interval, the number of relapses, re-resection, and chemotherapy use. Survival outcomes were assessed using Kaplan-Meier estimates and Cox proportional hazards models.

**Results:**

In the matched cohort (n = 98), progression-free survival (PFS) was significantly longer in the BNCT group compared with the control group (median 5.34 vs 3.70 months, hazard ratio [HR]: 0.54, *P* = .026), whereas overall survival (OS) was comparable (HR: 0.86, *P* = .524). The objective response rate was significantly higher in the BNCT group compared with the control group (67.4% vs 47.0%; *P* = .041). Multivariable analysis showed that the absence of callosal involvement (odds ratio [OR]: 0.10, *P* = .033) and higher minimum absorbed dose (OR: 1.09; *P* = .028) were independently associated with better BNCT response.

**Conclusions:**

Boron neutron capture therapy plus BEV was associated with higher objective response rate and improved PFS compared with BEV alone in patients with recurrent glioblastoma, although no OS difference was demonstrated. These findings support BNCT as a salvage strategy but warrant prospective validation.

Key Points

- First comparative study of BNCT plus BEV vs BEV alone in recurrent glioblastoma.
- BNCT plus BEV significantly prolongs progression-free survival compared with BEV alone.
- Absence of callosal involvement and higher minimum dose predict better BNCT responses.

Importance of the Study

This study addresses a critical gap by providing the first comparison of survival outcomes between recurrent glioblastoma patients treated with boron neutron capture therapy (BNCT) plus bevacizumab and those receiving bevacizumab alone. The addition of single-fraction BNCT was associated with higher response rate and significantly prolonged progression-free survival, although the overall survival (OS) impact was not demonstrated in this cohort. An exploratory subgroup analysis suggested a possible OS benefit in patients without callosal involvement, although this observation is limited by small sample size. Absence of callosal involvement and higher minimum absorbed dose were associated with better early response following BNCT. These findings help clarify the clinical impact of BNCT in this challenging disease and provide preliminary guidance for patient selection. Future studies are needed to validate these prognostic factors and further refine personalized application of BNCT in recurrent glioblastoma (GBM).

Glioblastoma is the most common and aggressive primary malignant brain tumor in adults. Despite a multimodal treatment approach comprising surgery, radiotherapy (RT), and temozolomide (TMZ), most patients inevitably experience recurrence within months.^1^ Therapeutic options for recurrent GBM are limited, and the prognosis remains poor.^2^^,^^3^ With advances in radiation techniques and systemic therapy, re-irradiation (re-RT) is increasingly considered for selected patients with localized recurrence.^4^ However, its use is limited by tumor size and the risk of severe toxicity in previously irradiated brain tissue. The short interval between initial treatment and recurrence further limits the feasibility of delivering an aggressive radiation dose.

Bevacizumab (BEV), a monoclonal antibody targeting vascular endothelial growth factor (VEGF), has been investigated to enhance the therapeutic ratio of re-RT.^5^ By mitigating vascular permeability and reducing peritumoral edema, BEV may help alleviate radiation necrosis and work synergistically with re-RT.^6^ The NRG Oncology/RTOG 1205 trial, a randomized phase II study, evaluated the addition of re-RT to BEV in recurrent GBM and demonstrated a significant improvement in 6-month progression-free survival (PFS).^7^ However, this did not translate into a significant overall survival (OS) advantage. These findings highlight the ongoing challenge of improving long-term outcomes in recurrent GBM and the need for novel therapeutic approaches.

Boron neutron capture therapy (BNCT) has emerged as an alternative re-RT strategy for recurrent GBM.^8^ The BNCT is a biologically targeted form of RT that exploits the preferential uptake of boron-10 (^10^B)-labeled amino acid derivatives, such as L-boronophenylalanine (BPA), by tumor cells. Once these boron-enriched tumor cells are irradiated with low-energy neutrons, the nuclear reaction occurs, producing high-energy alpha particles and lithium nuclei (^7^Li). The energy is deposited almost exclusively within 1 cell, thereby causing lethal damage only to the target cells while minimizing radiation exposure to surrounding healthy brain tissue. This selectivity depends on the overexpression of L-type amino acid transporter 1 (LAT1), the principal transporter of BPA, in GBM.^9^ Prior studies have reported that approximately 70% of viable tumors express LAT1,^10^ and its expression is associated with aggressive tumor behavior and poor prognosis in several cancer types.^11^^,^^12^

Given the biological advantages of BNCT, it has been a particularly attractive option for highly infiltrative tumors. Clinical studies have explored the application of BNCT in malignant gliomas and high-grade meningiomas.^8^^,^^13^^,^^14^ However, its survival advantage remains uncertain. Most existing studies have been either noncomparative or lacked appropriate control groups, making it difficult to establish BNCT’s therapeutic benefit. Furthermore, the heterogeneity among these studies—such as the inclusion of mixed populations with both grade 3 and grade 4 gliomas—poses an additional challenge in accurately assessing the clinical benefit of BNCT.

To address this gap, we conducted a retrospective review of a prospectively collected database to compare survival outcomes in patients with recurrent GBM treated with BEV-based systemic treatment alone versus those receiving additional BNCT. To our knowledge, this is the first study to evaluate the potential benefit of BNCT in recurrent GBM, providing valuable insights into its role as a salvage treatment for this challenging disease.

## Methods

### Study Population

Adult patients aged 20 years or older with histologically confirmed GBM (WHO 2021 classification) located in the cerebral hemispheres who experienced a first recurrence between January 1, 2015, and May 31, 2023, were evaluated for inclusion. First recurrence was defined as the initial radiographic evidence of tumor progression following completion of first-line treatment, assessed using the Response Assessment in Neuro-Oncology (RANO) criteria through multidisciplinary review.^15^ Magnetic resonance spectroscopy (MRS) and ^18^F-BPA PET (18F-boronophenylalanine positron emission tomography) were performed at the time of suspected recurrence to help distinguish true progression from treatment-related changes.

To ensure a homogeneous cohort for this analysis, patients were excluded if their tumors originated in the basal ganglia, thalamus, brainstem, or cerebellum, harbored an H3 K27M mutation, or if they had concomitant malignancies. All patients in the cohort had previously received standard first-line therapy consisting of maximal safe resection, followed by involved-field RT (60 Gy in 30 fractions) with concurrent and adjuvant TMZ.^1^^,^^16^ At first recurrence, patients with resectable disease typically underwent repeat resection, while those with unresectable or inoperable recurrence were treated with intravenous BEV at a dose of 10 mg/kg every 2 weeks, with or without BNCT and chemotherapy. The BNCT was considered for patients when ^18^F-BPA PET imaging shows adequate boron accumulation, defined as a tumor-to-normal tissue mean standardized uptake value (SUVmean) ratio of ≥ 2.5. Chemotherapy, when administered, consisted of alternating cycles of intravenous etoposide (100 mg/m^2^) plus carboplatin (450 mg/dose) and cyclophosphamide (800 mg/m^2^) plus vinblastine (5 mg/m^2^), continued until disease progression.

Patients were further excluded if they (1) did not receive salvage BEV; (2) received photon-based re-irradiation, tumor-treating fields, immunotherapy, investigational agents, or non-VEGF-targeted therapies; and (3) had more than 1 re-resection. The final study cohort was stratified into 2 groups: the control group that received BEV with or without chemotherapy, and the BNCT group that underwent an additional single-fraction BNCT. The study was approved by the Institutional Review Board of Taipei Veterans General Hospital (IRB No. 2020-11-002B, T-VGHTPE-52415) and informed consent was waived.

### Prognostic Variables

Clinical parameters were collected from our prospectively maintained database, including patient demographics, Karnofsky Performance Status (KPS), tumor characteristics, extent of resection, and salvage treatment types. The extent of resection was independently assessed by 2 board-certified neuroradiologists based on postoperative magnetic resonance imaging (MRI) within 72 hours of surgery. Resection was classified as gross total resection (GTR) (no residual enhancing tissue), near total resection (NTR) (<5 cm³ residual contrast-enhancing tissue), or subtotal resection (STR) (>5 cm³ residual contrast-enhancing tissue).^17^^,^^18^ No biopsy-only patients were included. Ventricular entry during craniotomy was defined as any intraoperative breach of the ependymal lining based on documentation in operative reports or, when not explicitly stated, assessed by early postoperative MRI.^19^ Corticosteroid use was categorized as perioperative only, short-term (<2 weeks post-surgery), or long-term (>2 weeks). Recurrent tumor volume was defined as the contrast-enhancing lesion on T1-weighted MRI. Callosal recurrence was defined as enhancement involving the corpus callosum, whereas ependymal recurrence was defined as enhancement along the ventricular lining. Callosal and ependymal involvement were recorded as discrete covariables because they reflect distinct patterns of tumor spread.

### Boron Neutron Capture Therapy

Reactor-based BNCT was performed at the Tsing Hua Open-Pool Reactor (THOR) using L-(4-^10^Borophenyl) alanine (L-^10^BPA) (Taiwan Biotech Company, Taiwan) as the boron carrier. Before BNCT, all patients underwent a ^18^F-BPA PET to assess boron uptake of different tissues. The boron uptake was quantified using the SUVmean. The SUVmean values were measured for the tumor, contralateral normal brain (reference for normal tissue, N), and heart ventricle (reference for blood pool, B). Tumor-to-normal tissue (T/N) and tumor-to-blood (T/B) ratios were then calculated by dividing the tumor SUVmean by the respective reference SUVmean values. A T/N ratio ≥ 2.5 was required for eligibility.^20^

During BNCT, patients were positioned based on the planned beam angles. The beam configuration and collimator size were selected based on tumor size and location. All treatments followed the same institutional protocol and dose constraints. Intravenous L-^10^BPA was administered at the rate of 200 mg/kg/h for the first 2 hours. Serum boron concentrations were measured using inductively coupled plasma atomic emission spectrometry (ICP-AES) at multiple time points: before infusion, 1 hour prior to irradiation, immediately before and after irradiation, and 30 minutes postirradiation. Neutron irradiation was initiated once the serum boron level reached 30 ppm. The infusion rate was then reduced to 100 mg/kg/h and continued throughout neutron irradiation. The total dose of L-^10^BPA administered was 500 mg/kg.^21^ Irradiation time was adjusted based on the T/N ratio and the blood boron concentration to achieve a mean GTV dose of 20 to 40 Gray-Equivalent (Gy-Eq) based on prior phase I/II studies.^21^ Gy-Eq was used to represent the biologically equivalent effect of radiation by multiplying the physical dose by the relative biological effectiveness (RBE) and compound biological effectiveness (CBE). In this study, the following values were applied: an RBE of 3.2 for thermal neutrons and an RBE of 1.0 for gamma radiation; an CBE values of 3.8 for tumors, 2.5 for skin, and 1.35 for nerves.^20^ The maximum and mean dose to normal brain tissue should be less than 12.5 and 3 Gy-Eq, respectively. Dose constraints were set at 10 Gy-Eq for the brainstem, 8 Gy-Eq for the optic nerves, 5.5 Gy-Eq for the inner ears, and 15 Gy-Eq for the skin and mucosa.^20^

### Endpoints

The primary endpoint was OS, defined as the time from BEV initiation (with or without BNCT) to death from any cause. This definition was chosen to reflect actual treatment exposure. To account for differences in disease course and treatment timing, the interval between the first recurrence and BEV initiation (with or without BNCT) was included as a covariate in the propensity score matching. Additionally, OS measured from the time of first recurrence was also evaluated as a secondary endpoint.

Other secondary endpoints included PFS, objective radiographic response, and treatment-related adverse events. Tumor response and progression were assessed using the RANO criteria via multidisciplinary review and independently evaluated by neuroradiologists.^18^ For patients treated with BNCT, objective response was evaluated 1-month posttreatment and classified as complete response (CR), partial response (PR), stable disease (SD), or progressive disease (PD). Magnetic resonance spectroscopy and ^18^F-BPA PET were incorporated in all patients to aid in distinguishing true tumor regression from pseudoresponse. Patterns of failure were classified as in-field (>80% overlap), marginal (20%-80% overlap), or distant (<20% overlap) based on the proportion of the recurrent tumor volume overlapping with the BNCT-irradiated volume.^22^ Treatment-related adverse events were graded using the Common Terminology Criteria for Adverse Events (CTCAE), version 5.0.

### Statistical Analysis

Continuous variables were presented as medians or means based on the Shapiro-Wilk test. Categorical variables were reported as counts and percentages. Comparisons between continuous and categorical variables were carried out using the Wilcoxon rank-sum test and Fisher exact test, respectively. To reduce confounding factors, patients were propensity matched 1:1 into the BNCT and control groups. Variables used for matching were as follows: age, performance status, recurrent tumor volume, recurrence-to-salvage-treatment interval, the number of relapses, re-resection, and chemotherapy use. Survival outcomes were estimated using the Kaplan-Meier method, and the between-group differences were assessed by the log-rank test. Hazard ratios (HRs) were estimated through univariate and multivariate Cox proportional hazards models. All statistical tests were 2-sided, with significance set at a *P* value < .05. Analyses were performed using R software version 4.2.1.

## Results

### Patient Characteristics

Of the 436 patients with recurrent GBM identified from our prospectively collected database, 171 met the inclusion criteria for analysis (Figure 1). Among them, 116 received BEV with or without chemotherapy (control group) and 55 received additional BNCT as part of salvage therapy (BNCT group). Baseline clinical and molecular characteristics were summarized in Table 1.

**Figure 1.** Consort diagram of patients assessed for eligibility. Abbreviations: CCRT, concurrent chemoradiotherapy; EGFR, epidermal growth factor receptor; TTF, tumor-treating fields.

**Table 1.** Patients’ characteristics before and after propensity score matching.

	Before matching			After matching		
	BNCT (*n* = 55)	Control (*n* = 116)	*P*	BNCT (*n* = 49)	Control (*n *= 49)	*P*
*Baseline characteristics*						
Gender (%)			.135			.686
Male	28 (50.9)	73 (62.9)		25 (51.0)	27 (55.1)	
Female	27 (49.1)	43 (37.1)		24 (49.0)	22 (44.9)	
IDH1 (%)			1			1
Wild type	55 (100.0)	116 (100.0)		49 (100.0)	49 (100.0)	
Mutant	0 (0.0)	0 (0.0)		0 (0.0)	0 (0.0)	
MGMT promoter (%)			.846			.885
Unmethylated	18 (32.7)	45 (38.8)		16 (32.6)	19 (38.8)	
Methylated	30 (54.6)	70 (60.3)		26 (53.1)	29 (59.2)	
Undetermined	7 (12.7)	1 (0.9)		7 (14.3)	1 (2.0)	
Ki-67 % (median, IQR)	40.0 (26.5-50.0)	50.0 (30.0-60.0)	.051	40.0 (27.0-47.5)	40.0 (30.0-50.0)	.167
Extent of initial resection (%)			.968			.303
Gross total resection	31 (56.4)	64 (55.2)		31 (56.4)	26 (53.1)	
Near total resection	14 (25.4)	29 (25.0)		14 (25.4)	9 (18.4)	
Subtotal resection	10 (18.2)	23 (19.8)		10 (18.2)	14 (28.6)	
Corticosteroids use (%)			.406			.453
Perioperative use	24 (43.6)	39 (33.6)		21 (42.9)	15 (30.6)	
Short-term (<2 weeks)	15 (27.3)	41 (35.3)		13 (26.5)	16 (32.7)	
Long-term (>2 weeks)	16 (29.1)	36 (31.0)		15 (30.6)	18 (36.7)	
Ventricular entry during craniotomy (%)			.484			.225
Yes	22 (40.0)	53 (45.7)		21 (42.9)	27 (55.1)	
No	33 (60.0)	63 (54.3)		28 (57.1)	22 (44.9)	
*First recurrence characteristics*						
Age, years (median, IQR)	51.0 (42.0-59.5)	60.0 (49.0-67.0)	<.001	52.0 (43.0-62.0)	53.0 (47.0-62.0)	.657
KPS (median, IQR)	80 (70-80)	70 (70-80)	.001	80 (70-80)	80 (70-80)	.280
Time to first recurrence, months (median, IQR)	8.0 (3.0-15.0)	6.0 (3.0-11.25)	.430	7.0 (3.0-15.0)	6.0 (4.0-10.0)	.517
Recurrent volume, mL (median, IQR)	47.6 (24.7∼70.7)	64.0 (31.5-106.4)	.047	48.0 (25.0-71.0)	58.1 (34.2-89.2)	.139
Callosal recurrence			.472			.541
Yes	25 (45.5)	46 (39.7)		23 (46.9)	20 (40.8)	
No	30 (54.5)	70 (60.3)		26 (53.1)	29 (59.2)	
Ependymal recurrence			.680			1.000
Yes	20 (36.4)	44 (37.9)		19 (38.8)	19 (38.8)	
No	35 (63.6)	72 (62.1)		30 (61.2)	30 (61.2)	
First recurrence-to-BEV interval, months (median, IQR)	1.74 (0.87-5.21)	1.18 (0.71-2.84)	.038	1.71 (0.84-4.37)	1.50 (0.87-3.23)	.787
No. of relapse at BEV initiation			.238			.223
First	26 (47.3)	66 (56.9)		24 (49.0)	30 (61.2)	
Second	29 (52.7)	50 (43.1)		25 (51.0)	19 (38.8)	
Re-resection (%)			.238			.223
Yes	29 (52.7)	50 (43.1)		25 (51.0)	19 (38.8)	
No	26 (47.3)	66 (56.9)		24 (49.0)	30 (61.2)	
Salvage chemotherapy (%)			<.001			.309
Yes	36 (65.5)	37 (31.9)		30 (61.2)	25 (51.0)	
No	19 (34.5)	79 (68.1)		19 (38.8)	24 (49.0)	
Salvage chemotherapy cycles until progression (median, IQR)	6 (5-8)	4 (2-6)	.021	6 (5-7)	5 (3-6)	.035

Abbreviations: BEV, bevacizumab; BNCT, boron neutron capture therapy; IQR, interquartile range; KPS, Karnofsky Performance Status.

At initial diagnosis, the median age of the cohort was 56 years (interquartile range [IQR]: 47-65); 59.1% (*n* = 101) were men, all cases were IDH-wild type, and 61.3% (*n* = 100) had MGMT promoter methylation. Initial tumor location, callosal involvement, ependymal involvement, and rates of gross total resection were similar between the 2 groups (all *P* > .05). At recurrence, the BNCT group had a younger age (median, 51.5 vs 60.5 years; *P* < .001), better performance status (median KPS, 80 vs 70; *P* = .001), and smaller tumor volume (median, 47.6 vs 64.0 mL; *P* = .047). Median time to first recurrence was comparable between groups (8.0 vs 6.5 months; *P* = .833), but the interval from first recurrence to initiation of salvage therapy was longer in the BNCT group (median, 1.74 vs 1.18 months; *P* = .038). Patterns of recurrence (callosal, ependymal, leptomeningeal) were similar across groups (all *P* > .05). Reoperation was performed in 79 patients (46.2%), with no significant differences between groups in reoperation rates, extent of re-resection, or changes in Ki-67 and MGMT status (all *P* > .05). Salvage chemotherapy was more frequently administered in the BNCT group (67.3% vs 31.9%; *P* < .001). The median follow-up was 20 months (IQR: 13-34 months).

### Overall Survival

**Figure 2.**
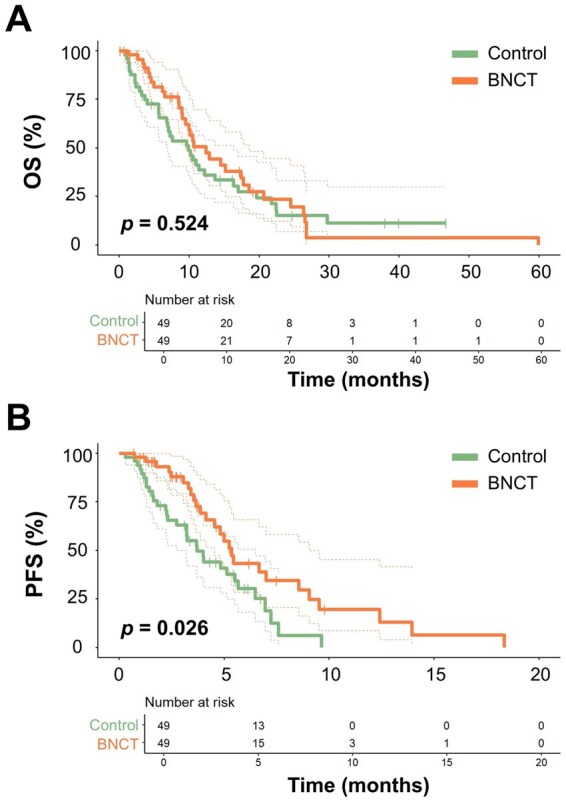
Kaplan-Meier survival analysis of BNCT and control group. (A) OS. (B) PFS. Abbreviations: BNCT, boron neutron capture therapy; OS, overall survival; PFS, progression-free survival.

To ensure a balanced comparison between the BNCT and control groups, 1:1 propensity score matching was performed using age, performance status, recurrent tumor volume, recurrence-to-salvage-treatment interval, the number of relapses, re-resection, and chemotherapy use. Because MGMT promoter methylation status can change over time, we did not include initial MGMT status in the propensity score model to avoid potential misclassification bias. After matching, 98 patients (49 per group) were included in the analysis, with baseline characteristics well balanced between groups (Table 1). The median OS after salvage treatment was 12.4 months (95% confidence interval [CI]: 9.5-18.5) in the BNCT group and 9.9 months (95% CI: 6.9-16.3) in the control group, a difference that was not statistically significant (HR: 0.86, 95% CI: 0.53-1.38; *P* = .524; Figure 2A). The 12-month OS rates were 50.6% (95% CI: 36.8%-69.7%) for the BNCT group and 41.7% (95% CI: 29.5%-56.6%) for the control group. Similarly, when OS was calculated from the time of first recurrence, no significant difference was observed (HR: 0.69; 95% CI: 0.44-1.11; *P* = .121).

In multivariable Cox regression, lower KPS, unmethylated MGMT promoter status, and ventricular entry during craniotomy were independently associated with shorter OS (Table 2). A post hoc analysis identified a statistically significant interaction between callosal involvement and BNCT (*P* = .041). Among patients without callosal recurrence, BNCT was associated with improved OS compared with the control group (HR: 0.51; 95% CI: 0.26-0.98; *P* = .044), whereas no OS benefit was observed in patients with callosal involvement (HR: 0.91; 95% CI: 0.43-1.97; *P* = .823). Overall, BNCT did not significantly improve OS in the matched cohort, but an exploratory post hoc analysis suggested a possible survival advantage in patients without callosal involvement. However, this observation should be interpreted cautiously given the small sample size.

**Table 2.** Cox regression analysis of overall survival in the propensity-matched cohort.

	Univariable analysis		Multivariable analysis	
	HR (95% CI)	*P*	HR (95% CI)	*P*
*Baseline characteristics*				
Female	0.93 (0.57-1.49)	.754		
Unmethylated MGMT promoter	1.72 (1.03-2.88)	.037	1.85 (1.09-3.14)	.022
Ki-67	1.01 (0.99-1.02)	.498		
Extent of initial resection				
Gross total resection	Reference			
Near total resection	1.25 (0.69-2.28)	.464		
Subtotal resection	1.34 (0.72-2.49)	.363		
Corticosteroid use				
Perioperative use	Reference			
Short-term (<2 weeks)	1.16 (0.62-2.14)	.644		
Long-term (>2 weeks)	1.23 (0.69-2.21)	.487		
Ventricular entry	2.00 (1.22-3.26)	.006	1.93 (1.07-3.50)	.030
*First recurrence characteristics*				
Age	0.99 (0.97-1.01)	.343		
KPS	0.96 (0.94-0.99)	.005	0.95 (0.92-0.99)	.018
Time to first recurrence	0.97 (0.93-0.99)	.033	0.97 (0.93-1.01)	.248
Recurrent volume	1.00 (0.99-1.01)	.371		
Callosal recurrence	1.20 (0.73-1.98)	.477		
Ependymal recurrence	1.78 (1.09-2.90)	.022	1.31 (0.73-2.37)	.366
Re-resection	1.26 (0.78-2.05)	.343		
Salvage chemotherapy	0.68 (0.42-1.11)	.121		
BNCT	0.78 (0.49-1.26)	.313		

Abbreviations: BNCT, boron neutron capture therapy; CI, confidence interval; HR, hazard ratio; KPS, Karnofsky Performance Status.

### Progression-Free Survival

Given the lack of a statistically significant OS benefit in the matched cohort, we next examined objective response and PFS as a measure of disease control following salvage treatment. In the propensity score–matched cohort, the objective response rate (ORR) was significantly higher in the BNCT group compared with the control group (67.4% vs 47.0%; *P* = .041). The CR rate was 8.2% in the BNCT group and 4.1% in the control group, respectively (Supplementary Table S1). The median PFS was 5.34 months (95% CI: 4.53-9.04 months) in the BNCT group compared with 3.70 months (95% CI: 2.75-6.47 months) in the control group (HR: 0.54; 95% CI: 0.31-0.93; *P* = .026; Figure 2B). The 6-month PFS rates were 43.4% (95% CI: 28.%-65.8%) in the BNCT group versus 30.5% (95% CI: 18.4%-49.1%) in the control group, respectively. These findings suggest that the addition of BNCT may provide better disease control compared with BEV alone.

### Toxicity

Treatment-related adverse events were assessed according to CTCAE version 5.0 (Supplementary Table S2). As anticipated, the BNCT group exhibited significantly higher rates of dermatologic toxicity. Radiation dermatitis occurred exclusively in the BNCT group (38.2% vs 0.0%; *P* < .001), although reactions were generally mild to moderate. Alopecia was also more common in the BNCT arm (98.2% vs 38.8%; *P* < .001), with cases in the control group likely attributable to chemotherapy.

Nausea and headache were numerically more frequent in the BNCT group, potentially due to cranial irradiation (transient increases in intracranial pressure) and the higher proportion of patients receiving chemotherapy; however, these symptoms were generally transient and manageable. While intracranial hemorrhage (all grades) occurred slightly more often with BNCT (9.1% vs 5.2%), severe events (grade ≥3) remained rare in both groups (1.8% vs 0.9%). Otherwise, no significant differences were observed in BEV-associated toxicities, including venous thromboembolism, hypertension, or proteinuria. Notably, no symptomatic radiation necrosis was identified in either group during the follow-up period.

### Identifying Key Predictors of BNCT Responders

We next evaluated factors associated with treatment response at 1 month after BNCT. In the BNCT cohort, the average absorbed dose was 29.16 ± 11.72 Gy-Eq. The maximum and minimum absorbed dose were 46.47 ± 16.92 and 11.90 ± 8.49 Gy-Eq, respectively. The median irradiated volume was 53.42 mL (IQR: 32.11-93.36 mL), and the median tumor depth was 6.30 cm (IQR: 5.43-7.27 cm). The median blood boron concentration during irradiation was 29.10 ppm (IQR: 25.43-34.75 ppm). The median T/N and T/B ratios were both 2.50 (IQR: 2.50-2.74). Univariable logistic regression identified several factors associated with BNCT responders (achieving CR or PR at 1-month post-BNCT), including the absence of callosal recurrence, smaller irradiated volume, shallower tumor depth, higher minimum absorbed dose, and older age (Table 3). In multivariable analysis, callosal involvement was an independent negative predictor of BNCT response (odds ratio [OR]: 0.10; *P* = .033), whereas higher minimum absorbed dose was associated with increased likelihood of response (OR: 1.09; *P* = .028). Taken together, these results highlight the importance of tumor location and delivered dose in patient selection for maximizing BNCT benefit.

**Table 3.** Factors associated with BNCT responders.

	Univariable analysis		Multivariable analysis	
	OR (95% CI)	*P*	OR (95% CI)	*P*
Age	1.07 (1.01-1.14)	.031	1.03 (0.9-1.18)	.671
KPS	1.03 (0.97-1.08)	.345		
Time to first recurrence	0.96 (0.91-1.02)	.191		
Callosal recurrence	0.12 (0.02-0.55)	.013	0.10 (0.01-0.66)	.033
Ependymal recurrence	1.37 (0.37-5.82)	.649		
Minimum absorbed dose	1.06 (1.02-1.12)	.011	1.09 (1.02-1.20)	.028
Irradiated volume	0.99 (0.98-0.99)	.038	0.99 (0.98-1.01)	.560
Tumor depth	0.55 (0.33-0.83)	.009	0.63 (0.34-1.08)	.112
Blood boron concentration	1.05 (0.95-1.17)	.375		
Tumor-to-normal ratio	0.71 (0.26-2.05)	.503		

Abbreviations: BNCT, boron neutron capture therapy; CI, confidence interval; KPS, Karnofsky Performance Status; OR, odds ratio.

A higher OR indicates a greater likelihood of achieving a treatment response.

## Discussion

Re-irradiation for recurrent GBM is challenging because prior radiation exposure to normal brain tissue often limits adequate dosing with conventional RT.^23^ The BNCT provides an alternative approach by selectively targeting boron-accumulating tumor cells while sparing surrounding tissue. Phase I/II trials have suggested potential benefits of BNCT in recurrent gliomas^24^; however, the lack of control groups in most studies makes it difficult to determine the true added value of BNCT.

In this retrospective, propensity score–matched analysis, we compared outcomes of patients receiving BNCT combined with BEV versus BEV alone. The addition of single-fraction BNCT was associated with significantly improved PFS (5.34 vs 3.70 months), although no significant OS difference was demonstrated between groups. These findings are consistent with the phase II NRG Oncology/RTOG 1205 trial, which also showed a PFS but no OS benefit when photon-based re-RT was added to BEV.^7^ Similarly, the recently presented GLIAA randomized trial reported no OS improvement when re-irradiation was guided with *O*-(2-[^18^F]fluoroethyl)-L-tyrosine (FET)-PET compared with MRI in recurrent GBM,^25^ underscoring the ongoing uncertainty regarding whether functionally targeted irradiation translates into survival benefit in the salvage setting.

Among various re-RT modalities, retrospective studies of photon-based re-RT have reported median OS ranging from 6.7 to 13 months, depending on the fractionation scheme: 7.5 to 13 months after stereotactic radiosurgery (15-20 Gy in a single fraction), 7.3 to 12.5 months after hypofractionated stereotactic RT (30 Gy in 5 fractions or 35 Gy in 10 fractions), and 6.7 to 11.5 months after conventional fractionation (36 Gy in 18 fractions).^23^ Outcomes with particle therapy were comparable, with proton therapy yielding median OS of 8.7 to 14.2 months,^26–28^ and carbon ion therapy achieving 8.0 to 10.5 months but with higher rates of grade ≥3 CNS toxicities.^29–31^ Notably, BNCT combined with BEV was well tolerated, with no radiation necrosis observed, and achieved comparable survival outcomes despite treating relatively large tumor volumes (median 53.4 vs 2.0-20.1 mL typically reported in earlier stereotactic radiosurgery studies). Our findings are consistent with prior studies combining BNCT and BEV, which similarly reported no radiation necrosis during follow-up.^32^ This likely reflects the favorable dose distribution of BNCT and the protective effects of BEV. However, we acknowledge that the sample size is limited and that subclinical necrosis cannot be entirely excluded. It should be noted that modern frameless and hypofractionated stereotactic radiosurgery techniques can also manage sizable recurrences with acceptable toxicity.^33^ Collectively, these advances in RT have expanded the therapeutic options for patients with larger-volume relapse.

In our cohort, KPS emerged as one of the most important predictors of OS in recurrent GBM. This is consistent with prior studies,^7^ supporting its use as a key criterion for patient selection in re-irradiation. In our exploratory subgroup analysis, the absence of callosal involvement was associated with a potential OS benefit after BNCT. One possible explanation is that callosal recurrence may reflect a more aggressive or infiltrative disease phenotype, which are often associated with multifocal progression and poor prognosis.^34^ Moreover, the limited penetration depth of thermal neutrons used in BNCT may reduce treatment efficacy for deep-seated lesions like those in the corpus callosum. Nevertheless, this was a post hoc observation based on a small subgroup and warrants validation in larger prospective cohorts.

Earlier BNCT studies without concurrent BEV reported shorter median OS: 7.0 months in the Finnish cohort,^35^ 8.7 months in the Swedish cohort,^36^ and 9.6 months in the Japanese cohort.^21^ A recent meta-analysis demonstrated that combining BEV with re-RT improved OS and reduced the risk of radiation necrosis in recurrent high-grade gliomas.^37^ Supporting this, the JG002 trial using accelerator-based BNCT plus BEV reported a median OS of 18.9 months,^38^ and Miyatake et al. observed a notably prolonged median OS of 21.4 months with the combinatorial strategy.^32^ Based on this evidence, all patients in our BNCT cohort received concurrent BEV. The relatively shorter OS in our cohort may be partly explained by differences in treatment parameters and disease burden. Specifically, our cohort received lower tumor doses (11.90-46.47 vs 39.4-75.6 Gy-Eq in Miyatake study) and had a larger median tumor volume (53.42 vs 35.1 mL), both of which may have contributed to reduced survival outcomes.

Identification of biomarkers for BNCT response remains an area of active investigation. Notably, neither serum boron concentration nor the tumor-to-normal boron uptake ratio was associated with treatment response at 1 month in our study. Although LAT1 serves as the primary transporter of BPA, findings from the EORTC 11001 trial demonstrated that histological overexpression of LAT1 does not correlate with boron uptake.^39^ This inconsistency likely arises when LAT1 is localized in the cytoplasm, where it is functionally inactive, or when the transporter is present but inactive. In our previous study, OS did not differ significantly between patients with T/N ratios ≥2.5 and those with lower ratios.^40^ One possible explanation is that high T/N ratios may reflect more aggressive tumor phenotypes—a hypothesis supported by a Japanese study reporting poorer post-BNCT survival among patients with elevated T/N ratios.^41^

Despite these advances, several practical challenges hinder the broader clinical adoption of BNCT.^42^ First, reactor-based BNCT requires access to specialized neutron sources and a highly coordinated, multidisciplinary team involving radiation oncologists, neurosurgeons, medical physicists, and nuclear engineers. Second, ^18^F-BPA PET imaging is not widely available and requires regulatory approval for production and use. Additionally, variability in national regulatory frameworks further complicates implementation. These factors limit the generalizability of BNCT and may explain why its clinical use is available only in a small number of centers worldwide. Increasing availability of accelerator-based BNCT systems may improve accessibility in the future.

To the best of our knowledge, this study represents the first and largest comparative analysis of BNCT in recurrent GBM. However, several limitations should be acknowledged. First, the retrospective nature of this study introduces inherent biases, despite the use of propensity score matching to reduce confounding. Second, the pseudoresponse effect of BEV may complicate radiographic interpretation, and the observed PFS-OS discrepancy warrants cautious interpretation. Although both groups received BEV, the lack of centralized imaging review may have affected the consistency of PFS evaluation. To minimize this concern, MRS and ^18^F-BPA PET were incorporated to help differentiate treatment-related changes. Notably, the BNCT group achieved a significantly higher ORR, suggesting that at least part of the radiographic improvement reflected true tumor control. Third, neurological symptoms and quality-of-life measures were not systematically collected, limiting our ability to directly correlate radiographic changes with clinical benefit. Finally, interpatient heterogeneity in BNCT delivery, boron biodistribution, and spatially variable LAT1 expression may have contributed to variability in treatment outcomes. Future prospective studies incorporating quality-of-life assessments, centralized imaging review, and biomarker-guided dose optimization are warranted to validate these findings and clarify the clinical impact of BNCT.

## Conclusions

Single-fraction BNCT combined with BEV was safe and well tolerated in recurrent GBM. The combination improved PFS compared with BEV alone, although an OS benefit was not demonstrated. Callosal involvement and absorbed dose were associated with treatment response. Prospective studies are warranted to optimize patient selection.

## Supplementary Material

vdag013_Supplementary_Data

## Data Availability

The de-identified data supporting the findings of this study are available from the corresponding author upon reasonable request.
