# Supplementary material for: Boron neutron capture therapy plus bevacizumab versus bevacizumab alone in recurrent glioblastoma: A propensity score–matched analysis
Source: Neurooncol Adv. 2026 Jan 23;8(1):vdag013. doi: 10.1093/noajnl/vdag013 (PMC13168812; doi:10.1093/noajnl/vdag013)
Supplement: vdag013_Supplementary_Data [file vdag013_supplementary_data.docx]

**Supplementary Material**

**Boron neutron capture therapy plus bevacizumab versus bevacizumab alone in recurrent glioblastoma: a propensity score–matched analysis**

Shuo-Fu Chen, Yi-Yen Lee, Chun-Yu Liu, Chun-Fu Lin, Sanford P. C. Hsu, Feng-Chi Chang, Chih-Chun Wu, Shih-Chieh Lin, Ko-Han Lin, Jia-Cheng Lee, Jinn-Jer Peir, Fong-In Chou, Hiroki Tanaka, Yu-Ming Liu, Yi-Wei Chen

***Table of Contents***

**Supplementary Table S1:** Response rates ……………………………...………….……………………………….……2

**Supplementary Table S2:** Treatment-related adverse events (CTCAE v5.0) ……………………………...…….………3

**Supplementary Table S1: Response rates**

|  | Before matching | |  | After matching | |
| --- | --- | --- | --- | --- | --- |
|  | **BNCT (n=55)** | **Control (n=116)** |  | **BNCT (n=49)** | **Control (n=49)** |
| Complete response, n (%) | 5 (9.1) | 3 (3.4) |  | 4 (8.2) | 2 (4.1) |
| Partial response, n (%) | 32 (58.2) | 44 (37.9) |  | 29 (59.2) | 21 (42.9) |
| Stable disease, n (%) | 15 (27.3) | 50 (43.1) |  | 13 (26.5) | 19 (38.8) |
| Progressive disease, n (%) | 3 (5.5) | 19 (16.3) |  | 3 (6.1) | 7 (14.3) |

**Abbreviations**: BNCT, boron neutron capture therapy.

**Supplementary Table S2: Treatment-related adverse events (CTCAE v5.0)**

|  | BNCT (n=55) | |  | Control (n=116) | |
| --- | --- | --- | --- | --- | --- |
|  | **Any grade (%)** | **Grade ≧ 3 (%)** |  | **Any grade (%)** | **Grade ≧ 3 (%)** |
| Acute toxicities |  |  |  |  |  |
| Radiation dermatitis | 21 (38.2) | 0 (0.0) |  | 0 (0.0) | 0 (0.0) |
| Alopecia | 54 (98.2) | 0 (0.0) |  | 45 (38.8) | 0 (0.0) |
| Nausea | 32 (58.2) | 2 (3.6) |  | 50 (43.1) | 2 (1.7) |
| Headache | 16 (29.1) | 1 (1.8) |  | 22 (19.0) | 1 (0.9) |
| Intracranial hemorrhage | 5 (9.1) | 1 (1.8) |  | 6 (5.2) | 1 (0.9) |
| Venous thromboembolism | 6 (10.9) | 4 (7.3) |  | 9 (7.8) | 5 (4.3) |
| Gastrointestinal perforation | 0 (0.0) | 0 (0.0) |  | 0 (0.0) | 0 (0.0) |
| Late toxicities |  |  |  |  |  |
| Hypertension | 16 (29.1) | 5 (9.1) |  | 38 (32.8) | 12 (10.3) |
| Proteinuria | 8 (14.5) | 1 (1.8) |  | 17 (14.7) | 1 (0.9) |
| Symptomatic radiation necrosis | 0 (0.0) | 0 (0.0) |  | 0 (0.0) | 0 (0.0) |

**Abbreviations**: CTCAE, Common Terminology Criteria for Adverse Events; BNCT, boron neutron capture therapy.
